# A self-reported measurement scale on a potential component of competency in the healthcare staff engaged in the prevention and control of non-communicable disease in Fiji

**DOI:** 10.1186/s12913-019-4695-8

**Published:** 2019-11-14

**Authors:** M. Ishikawa, M. Nomura, M. Miyoshi, N. Nishi, T. Yokoyama, H. Miura

**Affiliations:** 1 Department of Health Promotion, National Institute of Public Health, 2-3-6 Minami, Wako, Saitama, 351-0197 Japan; 2 Department of International Health and Collaboration, National Institute of Public Health, 3-6 Wako, Saitama, 351-0197 Japan; 3 Department of Nutrition, Faculty of Health Sciences, Aomori University of Health and Welfare, 58-1, Mase, Hamadate, Aomori, 030-8505 Japan; 4 International Center for Nutrition and Information, National Institutes of Biomedical Innovation, Health and Nutrition, 1-23-1 Toyama, Shinjuku-ku, Tokyo, 162-8636 Japan

**Keywords:** Healthcare staff, Competency, Non-communicable diseases, Social factors, Fiji

## Abstract

**Background:**

According to the World Health Organization, an estimated 80% or more deaths in Pacific island countries, including Fiji, were related to non-communicable diseases (NCDs). Although competency-based approaches have been effective for developing healthcare workers’ capabilities, there are only a few reports on competency scales of healthcare workers for NCD prevention. We aimed to develop a self-reported measurement scale on a potential component of competency in the healthcare staff engaged in the prevention and control of NCDs in Fiji.

**Methods:**

There were 378 Ministry of Health and Medical Services staff members working on NCD prevention and control in Fiji included in this study, which was a cross-sectional survey of social factors, working situation factors, and competency. Exploratory factor analysis was conducted to assess potential competency components, whereas Cronbach’s *α* coefficient and analysis of variance were used to assess the validity and reliability of the scale items, respectively. Multivariate regression analyses were conducted to analyze the respondents’ factor scores relative to social status and work situations.

**Results:**

The factor analysis revealed 16 items that identified competency in four work types: 1) work management, 2) monitoring and evaluation, 3) community partnership, and 4) community diagnosis. The monitoring and evaluation roles were related to ethnic background, community partnership was related to religion, and community diagnosis was related to academic qualifications.

**Conclusions:**

Based on the results, we developed a competency scale for the four work types. This scale can help healthcare workers engage in better management of residents with NCDs in Fiji.

## Background

In response to the 1995 Yanuca Island Declaration, issued by the World Health Organization (WHO), some Pacific island countries initiated non-communicable disease (NCD) prevention and control activities [1]. However, there has been no decrease in NCD prevalence [2]. The increases in the prevalence of NCDs, such as obesity, hypertension, and diabetes, in these countries have become a critical public health problem [3]. A recent WHO report estimated that 80% or more deaths in Pacific island countries were related to NCDs [4]. There is particular concern regarding the increase in deaths among those younger than 70 years. The cost burden of NCDs also puts pressure on government finances [4]. Therefore, it is important to develop the healthcare workers’ capacity to provide support for NCD prevention and control, as well as to promote these activities [5]. In Fiji, healthcare services have limited financial and human resources for managing NCDs. Healthcare workers typically manage multiple health problems, including maternal and child health, in addition to NCDs [6, 7].

In developing countries, including Fiji, promoting measures against NCDs have been carried out mainly through developing treatment methods, assuming that hospitals will improve the medical workers’ treatment or nursing skills [3, 8]. In reality, however, many people with early-onset NCD or those at high risk of having NCDs, such as individuals who are obese, are found within communities, not just in hospitals. Therefore, local community residents themselves must promote primary and secondary NCD prevention, and support from healthcare workers is important [9]. However, few studies have examined the approaches for improving healthcare workers’ capacity for community-based NCD prevention activities in developing countries.

There have been reports on the effectiveness of a competency-based approach for developing healthcare workers’ capabilities [6, 8, 10–15]. Competency indicates the combination of an individual’s motivation, personal values, character, cognitive skills, and knowledge, which incorporates a combination of these characteristics at work [6, 8, 10–16]. Competency indicators for public health workers have been developed in previous studies [11, 13]. These reports described important perspectives for health workers in communities, including community diagnosis and communication. These skills may also be essential competencies in the context of NCD prevention.

In Fiji, in 2007, a 14-item competency for nurses was developed to cover two major areas: core functional competency and generic competency [14]. This scale helped design a training plan that included on-the-job training to support the development of public health nurses. This scale emphasized medical care and competencies when working with patients (disease management) [17]. Recently, under the purview of Fiji’s Ministry of Health Medical Services (MOHMS), NCDs’ control and prevention have been focused on community efforts, with increased emphasis on prevention [18]. Healthcare workers need to improve their capabilities in activities emphasizing prevention through community health activities [7, 9].

Healthcare workers’ competencies may also be related to social factors, working environment, and employment conditions [5, 10]. However, workers’ competencies in relation to NCD prevention and control have not been investigated in Fiji. Therefore, the aims of this study were to develop a self-reported measurement scale on a potential component of competency of the healthcare staff engaged in the prevention and control of NCDs in Fiji and examine its association with social factors.

## Methods

### Participants and procedure

This study was conducted as part of the Project for Prevention and Control of Non-communicable Diseases under the Japan International Cooperation Agency and MOHMS [19].

Staff members in the Ministry of Health are assigned to manage, in each division, NCD prevention and control in health centers, hospitals, and/or nursing stations in Fiji’s Central Division. A list of 378 staff members, including nurses, dietitians, midwives, and health record managers, was created, and these staff members were included in the survey.

The survey was conducted from May to June 2016, and written informed consent was obtained from all the participants. Subdivisional public health nurses (SDHSs; health sisters) distributed an anonymous, hard-copy questionnaire in an envelope to the participants. The survey forms were either collected by the SDHSs or sent back directly to MOHMS.

Of the 378 staff members, 226 responded to the survey. Of the completed questionnaires, 179 had no missing values and were considered valid (response rate of 47.4%).

### Questionnaire

Questionnaire items were derived from NCD-related policies in Fiji, including (1) the National NCD Strategic Plan, (2) plan of action for nutrition, (3) Fiji’s wellness approach [18], (4) a WHO report [3], and (5) evidence from previous studies on human resource development or competency development of nurses, dietitians, or health workers in the field of public health (Additional file 1) [10, 11, 13–15, 20–27]. The questions covered various topics, such as competency in research, assessment, monitoring, guidance, and education; intervention management; working environment; and community development capacity. Each item covered one activity and was expressed clearly and concretely. We selected 42 items to reflect actual working activities in NCD prevention and control in Fiji.

In addition, social status and working situation factors were included in the survey to examine their association with competency. The social factors included sex, age, marital status, academic qualification (high school, diploma, bachelor’s degree, master’s degree, doctoral degree, or others), ethnic background (iTaukei, Fijian of Indian descent, or Fijian of other descent), and religion (Christian, Hindu, Buddhist, Muslim, or others). Working situation factors included facility type (hospital, health center, nursing station, senior citizens’ home, divisional office, subdivisional office, or others), professional qualification (nurse practitioner, registered nurse, dietitian, or others such as health record managers or health promotion personnel), and years of work experience, both overall and under MOHMS.

### Statistical analysis

A four-point Likert-type scale ranging from “strongly disagree” to “strongly agree” was used to measure all the competency items. An exploratory factor analysis was first conducted to identify the major work types based on the 42 items. To improve their interpretability, the Varimax method was used to rotate the identified factors by orthogonal transformation. Determining whether a factor should be retained was based on the natural interpretation of rotated factors with eigenvalues greater than 1 and a scree plot for the corresponding factors (i.e., a steep decline in the eigenvalue for the next factor; satisfied for four factors).

Two analyses were performed to confirm the items’ reliability and validity. In the first one, to assess reliability and internal consistency, Cronbach’s *α* coefficient was calculated for each factor pattern. However, extracting the factors was difficult because the *α* coefficients were not in the 0.70 to 0.95 range [28, 29] in the first analysis.

Therefore, in the next analysis, Pearson’s and Spearman’s correlation coefficients were used to examine the association between the total score and the score for each item; the correlation coefficients were almost the same. When there were differences in the results, Pearson’s correlation coefficient was used. Because factor analysis was parametric, Pearson coefficient was adopted for consistency. In instances where the Pearson’s correlation coefficient of two items was 0.7 or greater, one item from the pair was discarded, and factor analysis was conducted again. We performed such analyses with various patterns (exploratory analysis) [30].

As a result, we confirmed a four-factor solution, whose cumulative contribution reached 70% or greater (factor loadings of each item, ≥0.6). The factor contribution rate of the four factors is 74.2%, and the *α* coefficient of each factor was 0.85 to 0.92; thus, a total of 16 items (four for each factor) were extracted. Items that had a Pearson’s correlation coefficient of 0.4 or greater and *p* < 0.05 were identified. Each extracted factor was labeled a work type.

In addition, Student’s *t* test was used to verify the discriminatory power of the items, the differences between the mean values of the lowest category (Q1) and those of the highest category (Q4) for each item in the quartiles of total scores (hereinafter called good–poor analysis).

Analysis of variance was used to examine the associations between each factor score, social factors, and working situation. Among the working situation items, the duration of working experience under the MOHMS was divided into quarters (25, 50, and 75%). Finally, to examine the independent associations of the four-factor score with social status and working situations, multivariate regression analyses were performed, where the independent variables were selected by stepwise procedure with a *p* value of < 0.10 for entry and removal of all the variables of social status and working situation; sex and age were forced to enter into the model.

## Results

The study respondents’ characteristics are presented in Table 1. There were 21 (11.7%) men and 158 (88.3%) women, and 76.5% were married. The mean ± SD age of the respondents was 34.6 ± 7.8 years. Regarding ethnicity, nearly 65.4% were iTaukei and 31.5% were Fijian of Indian descent. For religion, 74.3% were Christian, and 21.2% were Hindu. Most (80.5%) had a diploma. Approximately one-fifth (19.0%) of the respondents worked in a hospital, and 65.9% worked in a health center; 83.8% were registered nurses and 3.9% were nurse practitioners (nurses with specialized training).

**Table 1:** Characteristics of the respondents (*n* = 179)

Item	n	%	SE
Sex			
Male	21	11.7	2.4
Female	158	88.3	2.4
Age			
mean ± SD	34.6	±7.8	0.6
-34 y	93	51.9	3.7
35–44 y	69	38.5	3.6
45- y	17	9.6	2.2
Marital status			
married	137	76.5	3.2
not married	42	23.5	3.2
Academic degree			
Certificate collage	4	2.2	1.1
Diploma	144	80.5	3.0
Bachelor degree	23	12.9	2.5
Master course of a graduate school	0	0.0	–
Doctoral course of a graduate school	0	0.0	–
Others	8	4.5	1.5
Ethnic background			
iTaukei	117	65.4	3.6
Fijian of Indian descent	56	31.3	3.5
Fijian of other descent	5	2.8	1.2
Refused-	1	0.6	0.6
Religion			
Christian	139	74.3	3.3
Hindi	38	21.2	3.1
Buddhism	5	2.8	1.2
Islam	1	0.6	0.6
Others	2	1.1	0.8
Refused	0	0	–
Working place			
health center	118	65.9	3.5
hospital	34	19.0	1.0
nursing station	12	6.7	1.9
diabetic hub centre	0	0.0	–
senior citizens home	1	0.6	0.6
divisional office	2	1.1	0.8
sub-divisional office	5	2.8	1.2
other	7	3.9	1.4
Health professional qualification			
nurse practitioner	7	3.91	3.6
registered nurse	150	83.8	2.8
dietitian	10	5.6	1.7
others	12	6.7	1.9
Duration of working experience under the MOHMS (months)			
mean ± SD	138.8	±90.7	6.8
	(11 years 6 months)		

*SD* standard deviation

*SE* standard error

The results of the scale measuring the healthcare staff’s competencies are summarized in Table 2. The results of the factor analysis identified four major work types that reflected professional experience: (1) work management, (2) monitoring and evaluation, (3) community partnership, and (4) community diagnosis, which accounted for 20.2, 20.0, 17.5, and 16.8% of the total variability, respectively, and 74.3% cumulatively.

**Table 2:** Factor loading matrix for competency among healthcare workers and reliability of the scale measurement for each item

	Factor 1	Factor 2	Factor 3	Factor 4	All			Good–Poor analysis	
Cronbach’s α coefficient	0.91	0.89	0.89	0.82	0.92	Item–Total analysis^a^		Difference between mean of Q1 and Q4	
Work type	Work management	Monitoring & evaluation	Community partnership	Community diagnosis	Commonality	Coefficient	p^a^		p^b^
The current status of one’s own work is well understood	0.878	0.119	0.155	0.139	0.829	0.565	< 0.0001	1.2	< 0.0001
Daily outcomes of one’s own work are well understood.	0.906	0.123	0.151	0.135	0.876	0.578	< 0.0001	1.2	< 0.0001
Purpose and significance of one’s own work are well understood	0.893	0.076	0.187	0.114	0.850	0.554	< 0.0001	1.1	< 0.0001
One’s own schedule/plan is appropriately managed	0.721	0.063	−0.014	0.365	0.658	0.487	< 0.0001	1.1	< 0.0001
Opportunities to participate in the programs/activities are equally provided to the community residents	0.139	0.840	0.226	0.181	0.808	0.659	< 0.0001	1.7	< 0.0001
Opportunities to participate in the programs/activities are equally provided to the relevant stakeholders, organizations, and institutions in the community	0.121	0.791	0.286	0.192	0.759	0.662	< 0.0001	1.6	< 0.0001
Progress of the programs/activities is appropriately reported to the community residents	0.118	0.787	0.252	0.190	0.732	0.637	< 0.0001	1.5	< 0.0001
Social resource and organizations are developed for implementation of the programs/activities	0.017	0.728	0.254	0.270	0.668	0.597	< 0.0001	1.4	< 0.0001
The relevant stakeholders, organizations, and institutions in the community to be collaborated with are selected for implementation of the programs/activities.	0.117	0.333	0.758	0.199	0.738	0.653	< 0.0001	1.5	< 0.0001
Consensus on necessity of the programs/activities is obtained with the relevant sections of governmental offices	0.217	0.289	0.795	0.196	0.800	0.697	< 0.0001	1.7	< 0.0001
Consensus on necessity of the programs/activities is obtained with the community residents	0.114	0.228	0.809	0.271	0.793	0.659	< 0.0001	1.4	< 0.0001
Roles for the programs/activities are coordinated with the relevant stakeholders, organizations, and institutions in the community	0.156	0.460	0.653	0.289	0.747	0.739	< 0.0001	1.6	< 0.0001
Current health problems are analyzed using epidemiological methods	0.202	0.225	0.154	0.612	0.490	0.542	< 0.0001	1.2	< 0.0001
Priority health problems which the government should work on are clearly identified	0.229	0.193	0.217	0.778	0.742	0.654	< 0.0001	1.5	< 0.0001
Solutions for health problems are investigated with a long-term viewpoint	0.047	0.209	0.243	0.752	0.670	0.572	< 0.0001	1.3	< 0.0001
National policies based on which the programs/activities are implemented, are well understood	0.292	0.204	0.208	0.742	0.721	0.667	< 0.0001	1.6	< 0.0001
Factor contribution	20.2	19.7	17.5	16.8					
Variance of factor	3.236	3.150	2.803	2.693					

^a^: p: Pearson correlation coefficient; ^b^: p: Student’s t-test

The analysis conducted to determine the reliability and validity of the competencies revealed that the Cronbach’s *α* coefficients were 0.91, 0.89, 0.89, and 0.82 for the four respective work types and 0.92 overall. The coefficients of all items were significant in the item–total analysis, and there were significant differences between the lowest (Q1) and highest (Q4) categories in the good–poor analysis.

The relationship between respondents’ characteristics and work type factor scores are summarized in Table 3. Work management was not related to any factors. In the monitoring and evaluation role, differences in terms of ethnic background were observed (*p* = 0.005). The highest scores were found for iTaukei workers, who had a mean score of 11.6 points, and the mean score of Fijians of Indian descent was 10.8 points.

**Table 3:** Relationship between respondents’ characteristics and factor score of work type

	Work management (Factor 1, 16 points)			Monitoring & evaluation (Factor 2, 16 points)			Community partnership (Factor 3, 16 points)			Community diagnosis (Factor 4, 16 points)		
	mean	SD	p	mean	SD	p	mean	SD	p	mean	SD	p
Sex												
Male	12.6	2.9	0.62	11.3	2.9	0.66	11.9	3.4	0.48	11.0	2.5	0.75
Female	12.3	2.9		11.2	2.9		11.6	2.7		10.9	2.7	
Age (year)												
≤ 34	12.6	2.7	0.37	11.3	2.7	0.88	11.3	2.6	0.21	11.2	2.4	0.24
35–44	12.0	3.1		11.2	3.1		11.9	2.8		10.5	3.0	
≥ 45	12.2	2.4		10.8	3.3		12.5	2.9		11.3	2.4	
Marital status												
Married	12.5	2.7	0.16	11.4	2.9	0.17	11.9	2.6	0.22	11.1	2.6	0.25
Not married	11.7	3.3		10.5	2.7		11.0	3.0		10.4	2.7	
Academic qualification												
Certificate college	14.3	2.1	0.13	11.0	4.8	0.07	13.5	3.3	0.07	13.0	2.9	0.007
Diploma	12.4	2.8		11.4	2.8		11.7	2.5		11.0	2.6	
Bachelor’s degree	11.2	3.4		9.7	3.0		10.8	3.7		9.3	2.7	
Others	12.8	2.8		12.8	2.2		13.4	2.4		12.4	2.4	
Ethnic background												
iTaukei	12.2	2.8	0.25	11.6	2.9	0.005	12.1	2.6	0.13	11.0	2.7	0.08
Fijian of Indian descent	12.8	2.8		10.8	2.7		11.0	2.8		11.1	2.5	
Fijian of other descent	11.8	2.8		7.6	1.1		11.0	2.9		8.2	2.4	
Religion												
Christian	12.3	2.7	0.27	11.5	2.9	0.11	12.1	2.5	0.06	11.0	2.7	0.93
Hindu	12.4	3.0		10.5	2.6		10.8	2.9		10.8	2.6	
Others	14.0	2.9		9.8	3.1		10.2	3.4		11.2	2.8	
Working place												
Health center	12.6	2.5	0.16	11.4	2.8	0.71	11.7	2.6	0.36	11.3	2.5	0.02
Hospital	12.1	3.5		10.8	3.1		11.4	3.3		10.1	2.8	
Nursing station	10.4	3.2		10.8	2.7		10.6	2.4		9.7	1.8	
Others	12.3	3.3		11.2	3.5		12.7	3.0		10.5	3.2	
Health professional qualification												
Nurse practitioner	12.1	2.7	0.22	12.7	2.7	0.48	13.4	2.2	0.31	11.7	2.1	0.51
Registered nurse	12.2	2.9		11.2	2.9		11.6	2.7		11.0	2.6	
Dietitian	14.2	2.0		10.5	3.0		11.2	3.2		10.6	3.8	
Others	12.2	3.3		11.4	3.1		12.1	3.5		9.8	2.3	
Duration of working experience under the MOHMS												
1–62 months (1 month−5 year 2 months)	12.5	2.9	0.86	11.1	2.6	0.86	11.3	2.6	0.04	11.0	2.4	0.85
63–128 months (5 year 2 months−10 year 7 months)	12.1	3.0		11.2	3.0		10.8	2.7		10.8	2.6	
129–179 months (10 year 8 months− 15 years)	12.5	2.9		11.1	3.0		12.0	2.7		11.1	3.0	
≥ 180 months (≥15 years)	12.2	2.8		11.5	3.0		12.5	2.7		10.7	2.7	

P data were analyzed using analysis of variance (ANOVA)

For community partnership, there were differences in the factor scores according to the duration of work experience for MOHMS (*p* = 0.04). There was an increase in community partnership score (average, 12.5 points) with an increase in length of work experience (≥15 years). For community diagnosis, there were differences according to academic qualifications (*p* = 0.007) and workplace (*p* = 0.02). The mean score for health center was 11.3 points, which was higher than the scores of other places.

The results of multiple regression analysis testing for the independent association of work types of competencies, social status, and working situations are presented in Table 4. Because the Pearson’s and Spearman’s correlation coefficient between age and duration of working experience under the MOHMS was 0.9 or more, the duration of working experience was removed, and age was included as an adjustment variable. Thus, work management was not related to any item. The monitoring and evaluation roles were related to ethnic background (*p* = 0.01). The parameter estimate of Fijians of other descent was lower than that of other ethnicities. Community partnership was related to religion (*p* = 0.03). The parameter estimate of Christians was higher than that of other religions. Community diagnosis was related to academic qualifications (*p* = 0.01). The parameter estimate of certificate college degree was higher than that of other academic degrees.

**Table 4:** Association between factor score of work type and respondents’ characteristics

			Work management (Factor 1)			Monitoring & evaluation (Factor 2)			Community partnership (Factor 3)			Community diagnosis (Factor 4)		
			Parameter estimate	SE	p	Parameter estimate	SE	p	Parameter estimate	SE	p	Parameter estimate	SE	p
Social status	Sex (male = 1; female = 2)		−0.88	0.67	0.29	−0.65	0.66	0.59	−0.75	0.63	0.36	−0.40	0.61	0.54
	Age (continuous variable; per + 10 years)		− 0.57	0.33	0.21	− 0.62	0.35	0.44	0.31	0.32	0.08	−0.29	0.33	0.40
	Marital status	Married	0 (ref.)		0.06	0 (ref.)		0.06		–			–	
		Not married	−1.00	0.52		−1.14	0.52			–			–	
	Academic degree	Certificate college		–			–			–		0 (ref.)		0.01
		Diploma		–			–			–		−2.14	1.36	
		Bachelor’s degree		–			–			–		−3.44	1.42	
		Others		–			–			–		−0.81	1.58	
	Ethnic background	iTaukei		–		0 (ref.)		0.01		–			–	
		Fijian of Indian descent		–		0.50	0.80			–			–	
		Fijian of other descent		–		−3.57	1.41			–			–	
	Religion	Christian		–		0 (ref.)		0.07	0 (ref.)		0.03		–	
		Hindu		–		−1.89	0.87		−1.12	0.51			–	
		Others		–		2.23	1.29		−1.88	1.11			–	
Working situation	Working place	Health center		–			–			–		0 (ref.)		0.06
		Hospital		–			–			–		−1.20	0.50	
		Nursing station		–			–			–		−1.29	0.81	
		Others		–			–			–		−0.47	0.74	
	Health Professional qualification			–			–			–			–	

The independent variables were selected by stepwise procedure with a *p* value of < 0.10 for entry and removal of all the variables of social status and working situation; sex and age were forced to enter into the model

Sex and age were forced to enter into the multivariable model

*SE*: standard error

-: the variable was not selected by stepwise procedure

## Discussion

### Characteristics of the four work types

The following four major work types were identified among the healthcare staff engaged in NCD prevention and control in central Fiji: 1) work management, 2) monitoring and evaluation, 3) community partnership, and 4) community diagnosis. The results also confirmed the reliability and validity of the competencies of the four work types. The importance of management skills, with regard to therapeutic and care relationships, the management of skills, knowledge, and emergency situations, together with quality improvement of risk management, is emphasized in the published competency framework for public health nurses in Fiji [14]. The findings provide an alternative scale for measuring the competencies of the healthcare staff working on NCDs, irrespective of career stage or clinical and public health backgrounds [10].

Similar categories have been identified in some previous studies of public health professionals’ competencies [9, 10, 25]. For example, the same skillsets were identified for the core competencies for such professionals in Canada, but in seven categories: (1) public health sciences; (2) assessment and analysis, policy, and program planning; (3) implementation and evaluation; (4) partnership, collaboration, and advocacy; (5) diversity and inclusiveness; (6) communication; and (7) leadership [10]. The need to strengthen systems for monitoring and evaluation and community diagnosis is highlighted in another study [20]. Community diagnosis work could be effectively broadened to cover areas affecting NCD prevention and control, such as primary healthcare reforms, moves toward universal health coverage, multisector collaboration, and social determinants of health [21, 31]. Some work may also be necessary to improve community diagnosis skill-based health promotion competencies in practice [22]. These efforts may help healthcare leaders improve planning for, monitoring, and evaluating NCDs [12].

### Association between work type and the characteristics of participants

The importance of considering social factors to improve healthcare workers’ competencies was revealed in this study, which found that the abilities to monitor and evaluate were related to ethnic background. In Fiji, the main ethnic groups are indigenous Fijians and immigrant Indians [32]. Historical policies continued the differences in language, religion, and culture between the two communities through residential and educational segregation and economic and political roles for different communities. The ethnic differences found in this study may relate to the ethnic characteristics and historical community values [32].

Challenges remain in identifying how to educate ethnically diverse groups of healthcare workers, including those from other countries, and improve the quality of NCD-related services. A potentially important element in NCD-related work is analyzing activity flow and the characteristics of healthcare workers who migrate, as is developing a system to integrate various ethnic groups [33, 34]. Therefore, developing teamwork (i.e., the ability to contribute to team activities through active participation and provide information to colleagues) among healthcare workers and communication skills (i.e., sharing relevant, accurate, and comprehensive information regarding client health through face-to-face and documented communications) may be important. More global competencies, including consideration of ethnic differences, should be identified in the future [34, 35].

This study has revealed that community partnership was related to religion. Several studies have shown that, in Fiji, the value of health was related to religious influences [36, 37]. Therefore, in creating a community network, it may be important to apply an approach that respects religious values and cooperation with related groups. Furthermore, in this study, community diagnosis was found to be related to academic qualifications. Strengthening the knowledge and skills related to the daily monitoring of community residents, developing the capacity and ability to support a system for strategic community diagnosis and monitoring, and implementing appropriate data collection for effective intervention are important for providing effective community diagnosis [20, 38]. For these activities, training may need to be aligned with academic qualifications [39].

Consideration must be made on the proper application of these competency scores for healthcare workers and for improving the management of NCD patients in Fiji. The self-assessment of NCD prevention and control work via this competency scale is possible, and the competency score suggests the feasibility of analyzing the strengths and weaknesses of work concerning NCDs. This analysis, in turn, can help determine the status of the NCD-related work capacity of healthcare workers and plan specific human resource development strategies for reducing the numbers of NCD patients. Such analyses may also contribute to other Pacific countries struggling with NCDs [3].

The study has some limitations. First, no causal relationships could be inferred because of the cross-sectional study design; the observations could be accounted for by reverse causation or simple correlation. Second, the response rate was 47.4%. The healthcare workers who did not answer may not have had sufficient time to answer the questionnaires during their work hours, and because the surveys were anonymous, we were unable to check response status before collecting the surveys. A follow-up study using face-to-face interviews with participants should be conducted. Also, non-respondents may have had difficulty in answering the questionnaire’s socioeconomic items. The results might have been affected by non-responders. A survey method that includes follow-up research on interviews with ethical considerations for the participants should be used in the future.

Third, the factor analysis was limited, in terms of subjectivity, in determining and labeling work types that can also be applied to other populations. Although the exploratory analysis was conducted using 42 items for 179 respondents, this represents a ratio between the number of items and number of respondents of approximately 1:4.5, which may be considered as a lower threshold of acceptability [28, 30]. It is important to conduct examinations using rest–retest (confirmation of reproducibility by practice) in the future [30].

However, despite these limitations, this study found a close relationship between competency in particular work types and some social factors in Fiji. To improve the competency ability of healthcare workers engaged in NCD prevention and control, it might be important to create a human resources development curriculum that considers the relationship between work types and socioeconomic factors.

## Conclusion

In this study, we developed a scale for measuring the competence of the healthcare staff engaged in preventing and controlling NCDs, specifically in Fiji. The scale covers four types of work: work management, monitoring and evaluation, community partnership, and community diagnosis. Notably, relationships were found between monitoring and evaluation and ethnic background, between community partnership and religion, and between community diagnosis and academic qualifications. This scale will help healthcare workers better manage residents with NCDs and can help plan specific human resource development strategies for reducing NCD prevalence in Fiji.

## Supplementary information

**Additional file 1.** Questionnaire on competencies required for working activities in prevention and control of non-communicable diseases (NCDs) (Additional file 1).

## Data Availability

Further information about the data used and analyzed during the study may be available from the Fiji’s Ministry of Health Medical Services (MOHMS) on reasonable request.

## Abbreviations

SD: Standard deviation
SE: Standard error
