# Supplementary material for: A self-reported measurement scale on a potential component of competency in the healthcare staff engaged in the prevention and control of non-communicable disease in Fiji
Source: BMC Health Serv Res. 2019 Nov 14;19:838. doi: 10.1186/s12913-019-4695-8 (PMC6857309; doi:10.1186/s12913-019-4695-8)
Supplement: Supplementary file 1 — Additional file 1. Questionnaire on competencies required for working activities in prevention and control of non-communicable diseases (NCDs) (Additional file 1). [file 12913_2019_4695_MOESM1_ESM.docx]

**Questionnaire on competencies required for working activities**

**in prevention and control of non-communicable diseases (NCDs)**

Please indicate the extent of your agreement or disagreement with each of the following statements by marking the appropriate circle to the right of each statement.

Please use the following 4-point scale (a higher number on the scale indicates more agreement)

**○**

| ***1------------2------------3------------4***  ***Strongly Disagree Strongly Agree*** |
| --- |

| Question | | Response |
| --- | --- | --- |
| 1^†^ | The current status of one’s own work is well understood. | 1 -----2-----3-----4 |
| 2^†^ | Daily outcomes of one’s own work are well understood. | 1 -----2-----3-----4 |
| 3^†^ | Purpose and significance of one’s own work are well understood. | 1 -----2-----3-----4 |
| 4^†^ | One’s own schedule/plan is appropriately managed | 1 -----2-----3-----4 |
| 5 | Contents of one’s work are appropriately taken over, on the occasion of job transfer. | 1 -----2-----3-----4 |
| 6 | Needs among the community residents are well grasped. | 1 -----2-----3-----4 |
| 7 | Information on the health problem is appropriately collected. | 1 -----2-----3-----4 |
| 8 | Information on the health problems is appropriately used. | 1 -----2-----3-----4 |
| 9^†^ | Current health problem is analyzed using epidemiological methods | 1 -----2-----3-----4 |
| 10 | Current health problem is assessed from various directions. | 1 -----2-----3-----4 |
| 11^†^ | Priorities of health problem on which the government should work are clearly identified. | 1 -----2-----3-----4 |
| 12^†^ | Solutions for health problem are investigated in a long-term viewpoint. | 1 -----2-----3-----4 |
| 13^†^ | National policies based on which the programs/activities are implemented are well understood. | 1 -----2-----3-----4 |
| 14 | Solutions for health problem are investigated based on the national policies. | 1 -----2-----3-----4 |
| 15 | Programs/activities are planned based on the needs among residents. | 1 -----2-----3-----4 |
| 16 | Health problems to be solved are shared within one’s present working facility | 1 -----2-----3-----4 |
| 17 | Health problems to be solved are shared with the relevant sections of governmental offices. | 1 -----2-----3-----4 |
| 18 | Health problems to be solved are shared with the community residents. | 1 -----2-----3-----4 |
| 19 | Health problems to be solved are shared with the relevant stakeholders, organizations and institutions in the community. | 1 -----2-----3-----4 |
| 20 | The relevant sections of governmental offices to be collaborated with are selected for implementation of the programs/activities | 1 -----2-----3-----4 |
| 21 | The community residents to be collaborated with are selected for implementation of the programs/activities | 1 -----2-----3-----4 |
| 22^†^ | The relevant stakeholders, organizations and institutions in the community to be collaborated with are selected for implementation of the programs/activities | 1 -----2-----3-----4 |
| 23 | Consensus on necessity of the programs/activities is obtained within one’s present working facility. | 1 -----2-----3-----4 |
| 24^†^ | Consensus on necessity of the programs/activities is obtained with the relevant sections of governmental offices. | 1 -----2-----3-----4 |
| 25^†^ | Consensus on necessity of the programs/activities is obtained with the community residents. | 1 -----2-----3-----4 |
| 26 | Consensus on necessity of the programs/activities is obtained with the relevant stakeholders, organizations and institutions in the community. | 1 -----2-----3-----4 |
| 27 | Roles for the programs/activities are coordinated within one’s present working facility. | 1 -----2-----3-----4 |
| 28 | Roles for the programs/activities are coordinated with the relevant sections of governmental offices. | 1 -----2-----3-----4 |
| 29 | Roles for the programs/activities are coordinated with the community residents. | 1 -----2-----3-----4 |
| 30^†^ | Roles for the programs/activities are coordinated with the relevant stakeholders, organizations and institutions in the community. | 1 -----2-----3-----4 |
| 31^†^ | Opportunities to participate in the programs/activities are equally provided to the community residents. | 1 -----2-----3-----4 |
| 32^†^ | Opportunities to participate in the programs/activities are equally provided to the relevant stakeholders, organizations and institutions in the community. | 1 -----2-----3-----4 |
| 33 | Discussion and supervision are well performed in collaboration with lower-level facilities. | 1 -----2-----3-----4 |
| 34 | Based on 33, the current status and problems of lower-level facilities are appropriately grasped. | 1 -----2-----3-----4 |
| 35 | Progress of the programs/activities is appropriately reported within one’s present working facility. | 1 -----2-----3-----4 |
| 36 | Progress of the programs/activities is appropriately reported to the relevant sections of governmental offices (e.g. higher-level facilities). | 1 -----2-----3-----4 |
| 37^†^ | Progress of the programs/activities is appropriately reported to the community residents. | 1 -----2-----3-----4 |
| 38 | Progress of the programs/activities is appropriately reported to the relevant stakeholders, organizations and institutions in the community. | 1 -----2-----3-----4 |
| 39^†^ | Social resource and organizations are developed for implementation of the programs/activities. | 1 -----2-----3-----4 |
| 40 | Changes of health status in the community are appropriately evaluated. | 1 -----2-----3-----4 |
| 41 | Results of the evaluation are fed back to planning of the programs/activities. | 1 -----2-----3-----4 |
| 42 | Leadership is demonstrated in planning/implementation of the programs/activities. | 1 -----2-----3-----4 |

†: The item extracted in this study
